# Pain-Related Abnormal Neuronal Synchronization of the Nucleus Accumbens in Parkinson’s Disease

**DOI:** 10.3390/brainsci12010084

**Published:** 2022-01-07

**Authors:** Kaoru Kinugawa, Tomoo Mano, Yuya Yamatani, Toshiteru Miyasaka, Hiroshi Kataoka, Kazuma Sugie

**Affiliations:** 1 Department of Neurology, Nara Medical University, 840 Shijo-cho, Kashihara 634-8521, Japan; kinugawa_kaoru@naramed-u.ac.jp (K.K.); hk55@naramed-u.ac.jp (H.K.); ksugie@naramed-u.ac.jp (K.S.); 2 Division of Central Radiology, Nara Medical University Hospital, 840 Shijo-cho, Kashihara 634-8521, Japan; y-yama@naramed-u.ac.jp; 3 Department of Radiology, Nara Medical University, 840 Shijo-cho, Kashihara 634-8521, Japan; tmiyasaka@naramed-u.ac.jp

**Keywords:** pain, Parkinson’s disease, resting-state functional MRI, functional connectivity, nucleus accumbens, mesolimbic pathway

## Abstract

Patients with Parkinson’s disease (PD) often experience pain, which fluctuates in “on” and “off” states, but the underlying mechanism is unclear. The nucleus accumbens (NAc) is a central component of the mesolimbic dopaminergic pathway involved in pain processing. We conducted resting-state functional magnetic resonance imaging (rsfMRI) analysis to explore the relationship between the neuronal synchronization of NAc with pain-related brain regions and pain intensity in “on” and “off” states. We assessed 23 patients with sporadic PD based on rsfMRI and pain intensity using the revised Short-Form McGill Pain Questionnaire. Patients with PD displayed higher pain intensity scores in the “off” state than in the “on” state. The pain intensity in the “off” state was substantially correlated with the functional connectivity (FC) between the NAc and primary motor/sensory cortices and contralateral NAc. Changes in pain intensity from the “on” to “off” state displayed correlations with those between the right (rNA) and left NAc (lNAc) and the right precentral gyrus (rPreCG) /right insular cortex (rIC) from the “off” to “on” state. Aberrant bilateral NAc and rNAc–rPreCG/rIC FC in the “off” state were closely related to pain symptoms developed from the “on” to “off” states. These results suggest that the NAc in the mesolimbic pathway is related to pain in PD and may help understand the mechanism of pain development in patients with PD.

## 1. Introduction

In recent years, researchers have gained interest in non-motor symptoms of Parkinson’s disease (PD). Several non-motor symptoms, including pain, affect patients with PD [1]. Pain is present in approximately two-thirds of patients with PD and exerts a major impact on their quality of life. Most patients with PD perceive pain as the most challenging symptom at all stages of the disease [2]. Pain symptoms tend to worsen in the “off” state [3]. Oral antiparkinsonian drugs particularly affect the fluctuation of pain, thus suggesting the dopaminergic fluctuation associated with pain in PD [4]. This pain is often under-recognized and undertreated, which can be attributed to inadequate understanding of the associated mechanism.

Several pathways involved in sensory processing modulate pain [5]. These pathways are altered across the “on” and “off” states in PD, and an alteration of the pathways is closely related to pain processing in the brain [6,7,8]. The mesolimbic pathway is associated with reward and motivation and plays an important role in the perception and modulation of pain [5]. The nucleus accumbens (NAc) is a central component of the mesolimbic pathway and is connected to pain-related brain regions [5]. In addition, connectivity between the NAc and other brain regions substantially contribute to the sensory and emotional aspects of pain sensation and its modulation in neuropsychiatric disorders, including PD [5,9].

Abnormal neuronal synchronization in brain networks is related to the development of pain in PD [8,10]. We hypothesized that changes in functional connectivity (FC) of NAc in patients with PD are related to the fluctuation of pain from the “on” to “off” states. To explore the mechanism of pain related to the “on” and “off” state fluctuations, we aimed to investigate the alterations of FC in brain networks related to NAc using resting-state functional MRI (rsfMRI) analysis. We assessed the severity and characteristics of pain experienced by PD patients using the Japanese version of the revised short-form McGill Pain Questionnaire 2 (SF-MPQ-2) [11]. We intended to explore the relationship between the severity and characteristics of pain and FC from the “on” to “off” states in patients with PD.

## 2. Materials and Methods

### 2.1. Patients

We enrolled consecutive patients with sporadic PD who visited Nara Medical University Hospital (Nara, Japan) between June 2020 and May 2021. A total of 23 patients fulfilled the inclusion and exclusion criteria. The major inclusion criteria were: (1) presenting confirmed PD and meeting the UK PD Society Brain Bank criteria [12]; (2) being 20–90 years old; (3) presenting the wearing-off phenomenon; and (4) absence of significant lesions on MRI. The exclusion criteria were: (1) having other known causes of pain (e.g., spinal cord disease, orthopedic disease, and peripheral neuropathy); (2) having undergone operations such as deep brain stimulation; (3) presence of severe cognitive dysfunction (Mini-Mental State Examination [MMSE] < 10); (4) presence of severe mental disorders; and (5) not being considered appropriate for participation in the trial. We excluded patients with claustrophobia and those with involuntary movements who could not remain still during MRI acquisition. Ethical approval for this study was obtained from the Nara Medical University Clinical Research Ethics Board. Written informed consent was obtained from all patients. All study procedures were performed in accordance with the ethical standards of the aforementioned institutional research committee and adhered to the principles of the Declaration of Helsinki and the Ethical Guidelines for Medical and Health Research Involving Human Subjects in Japan.

### 2.2. Clinical Assessments and Study Design

Patients with PD were observed for one week prior to MRI examinations and were assessed in the “on” and “off” states, which correspond to states when dopamine replacement therapy is effective and symptoms are controlled and when the treatment effect wears off and symptoms re-emerge. The MRI examinations were performed on each patient during both the “on” and “off” states over two testing days. Patients were assessed using the Movement Disorder Society Unified Parkinson’s Disease Rating Scale (MDS-UPDRS) part III, Numerical Rating Scale (NRS; 0 to 10 points), and SF-MPQ-2 at the time of the MRI examination [11,13,14]. SF-MPQ-2 total scores (22 items, 0 to 220 points) were divided into four subscales: continuous pain (six items, 0 to 60 points), intermittent pain (six items, 0 to 60 points), neuropathic pain (six items, 0 to 60 points), and affective descriptors (four items, 0 to 40 points) [11]. The differences in the SF-MPQ-2 total and subscale scores of each patient between the “on” and “off” states were calculated. The cognitive and neurological assessment of patients was performed using the MMSE, Frontal Assessment Battery, and the MDS-UPDRS (part I, II, IV) on separate days [15]. The levodopa-equivalent daily dose was calculated [16]. The clinical features of the patients are summarized in Table 1.

### 2.3. MRI Acquisition

Patients underwent MRI examination on the two testing days: one testing day was an “on” day and the other one was an “off” day. The MRI examinations were preceded by a clinical assessment based on the MDS-UPDRS Part III, NRS, and SF-MPQ-2. Functional and structural MRI data were acquired using a 3-Tesla MRI scanner (MAGNETOM Skyra, Siemens Healthcare) with a 20- or 32-channel head coil at Nara Medical University Hospital. A total of four patients received MRI scans with a 20-channel head coil because of postural instability. The functional images were acquired using a gradient-echo echo-planar pulse sequence sensitive to blood oxygen level dependent (BOLD) contrast with the following parameters: repetition time (TR) = 1190 ms, echo time (TE) = 31 ms, matrix size = 64 × 64 mm^2^, flip angle (FA) = 90°, field of view (FOV) = 212 × 212 mm^2^, slice thickness = 3.2 mm, and voxel size = 3.3 × 3.3 × 3.2 mm^3^, for a total of 40 slices. Two runs, each with 245 volumes, were performed for each patient. The T1-weighted structural images were acquired with the following parameters: TR = 1900 ms, TE = 2.75 ms, inversion time = 900 ms, matrix size = 256 × 256 mm^2^, FA = 9°, FOV = 256 × 256 mm^2^, slice thickness = 1 mm, and voxel size = 1.0 × 1.0 × 1.0 mm^3^, for a total of 192 slices. The patients were instructed to keep their eyes centered on the fixation cross and to not think about anything during the acquisition phase [17].

### 2.4. Functional MRI Data Preprocessing

The MRI data were preprocessed using the Statistical Parametric Mapping software, version 12 (SPM12), and the CONN toolbox, version 17, in MATLAB (version R2020a, MathWorks, Natick, MA, USA) [18]. The preprocessing of functional and structural data was performed following the default preprocessing pipeline in the CONN toolbox. The initial ten scans of functional images were removed to eliminate equilibration effects. The functional images were realigned and unwarped; slice-timing corrected (in ascending order); segmented into gray matter (GM), white matter (WM), and cerebrospinal fluid (CSF); spatially normalized to the Montreal Neurological Institute (MNI)-space (Montreal, Canada); screened for outliers (artifact detection tools [ART]-based scrubbing); and smoothed to 8 mm full width at half maximum Gaussian kernel. To eliminate the influence of head motion and artifacts during the experiment, we identified outlier time points in the motion parameters and global signal intensity using ART, which is included in the CONN toolbox. The structural images were also segmented into GM, WM, and CSF and normalized to the MNI-space. Subsequently, denoising was performed on the functional images by default, including band-pass filtering (0.008 to 0.09 Hz), linear detrending, and nuisance regression of motion related components via the component-based noise correction method.

### 2.5. Functional Connectivity Analysis

The resting-state FC analysis was performed using a seed-based approach using the CONN toolbox. Region of interests (ROIs) were selected from the FMRIB Software Library Harvard-Oxford cortical and subcortical structural atlases [18]. Based on previous literature, we chose 22 ROIs known to be associated with pain: bilateral NAc, bilateral globus pallidus, bilateral thalamus, bilateral insular cortex, bilateral amygdala, bilateral posterior parahippocampal gyrus, bilateral hippocampus, bilateral anterior parahippocampal gyrus, bilateral postcentral gyrus, bilateral precentral gyrus, anterior cingulate gyrus, and brainstem [8,19,20,21,22]. The bilateral NAc ROIs were used as seed regions, and the other ROIs were used as target regions for further analysis (Table S1). In the first-level analysis, the mean BOLD timeseries from each ROI was estimated by averaging the timeseries of all voxels at each ROI. Bivariate correlation coefficients were calculated between each pair of the BOLD timeseries from the selected ROIs. Fisher’s transformation was applied to the resulting coefficient values. Each Fisher’s-transformed coefficient value represented the FC value between two brain regions. The difference in FC values measuring ROI-to-ROI connectivity was calculated between “on” and “off” states (the subtraction of FC values in the “off” state from those in the “on” state). Correlations between the SF-MPQ-2 scores and FC values were assessed using the Pearson’s correlation coefficient.

### 2.6. Statistical Analysis

All statistics were performed using MATLAB version R2020a. The means of the obtained values were compared between the “on” and “off” states using the paired two-sample *t*-test. Correlations between scores on the clinical assessments and FC values were assessed using the Pearson’s correlation coefficient. The Shapiro-Wilk test was used to assess the normality of data distribution. *p*-values of <0.05 were considered statistically significant, and a non-significant trend was established for a *p*-value between 0.05–0.15. A correlation was considered strong if the coefficient (*r*) was >0.40.

## 3. Results

### 3.1. Demographic and Clinical Characteristics

Twenty-three patients with PD (12 male and 11 female) were enrolled in this study. The demographic characteristics and clinical features of the 23 patients with PD are summarized in Table 1. Patients displayed significantly higher MDS-UPDRS Part III scores in the “off” state than in the “on” state (*p* < 0.05). There was a non-significant trend for higher NRS scores in the “off” state than in the “on” state (*p* = 0.068). Moreover, patients displayed higher SF-MPQ-2 total and affective subscale scores in the “off” state than in the “on” state (*p* < 0.05). There was a non-significant trend for higher continuous, intermittent, and neuropathic subscale scores of SF-MPQ-2 in the “off” state than in the “on” state (*p* = 0.11, 0.12, and 0.12, respectively). These results indicate that patients with PD experience pain, as well as motor disability, during the “off” state in their daily life.

### 3.2. Relationship between FC of the NAc and Pain Intensity in “on” and “off” States

**Figure 1:**
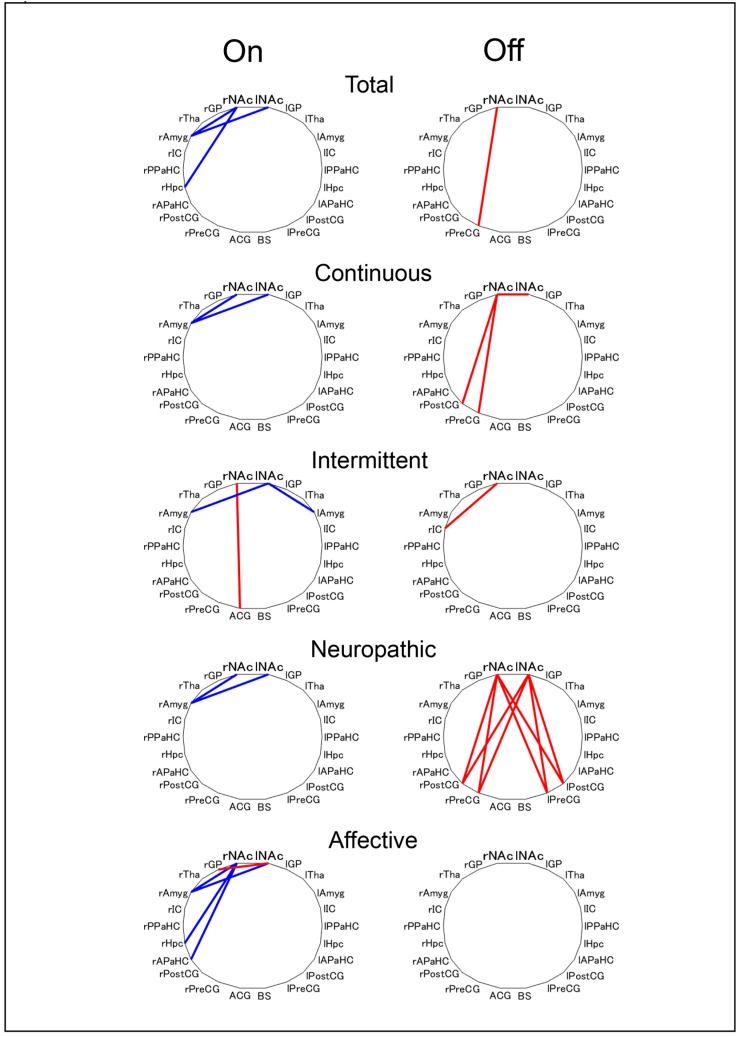
Characteristics of correlation coefficient differences between the “on” and “off” states. Blue lines indicate a significantly negative correlation (<−0.4), and red lines indicate a significantly positive correlation (>0.4). SF-MPQ-2: The Japanese version of the revised short-form McGill Pain Questionnaire 2; FC: functional connectivity; r: right; l: left; NAc: Nucleus Accumbens; GP: Globus Pallidus; Tha: Thalamus; Amyg: Amygdala; IC: Insular Cortex; PPaHC: Posterior Parahippocampal Gyrus; Hpc: Hippocampus; APaHC: Anterior Parahippocampal Gyrus; PostCG: Postcentral Gyrus; PreCG: Precentral Gyrus; ACG: Anterior Cingulate Gyrus; BS: Brainstem.

We explored the relationship between FC in brain networks and pain intensity in patients with PD. Using the bilateral NAc as seed regions, the seed-based FC analysis was conducted. In the “off” state, the SF-MPQ-2 total scores displayed a positive correlation (*r* = 0.40) with FC between the right NAc (rNAc) and right precentral gyrus (rPreCG) (Figure 1). Dividing the total SF-MPQ-2 scores into four subscales, the continuous subscale scores displayed a positive correlation with FCs of the rNAc with rPreCG (*r* = 0.44), right postcentral gyrus (rPostCG; *r* = 0.43), and left NAc (lNAc; *r* = 0.41), whereas neuropathic subscale scores displayed a positive correlation with FCs of bilateral NAc with the bilateral PreCG and PostCG (Figure 1). In contrast, in the “on” state, there were significant correlations between the SF-MPQ-2 scores and FCs, which were not found in the “off” state. For example, the SF-MPQ-2 total, continuous, and neuropathic subscale scores displayed a negative correlation with the FCs of the bilateral NAc with the right amygdala (Figure 1). The correlation of FC and pain intensity was altered between “on” and “off” states in patients with PD.

### 3.3. Relationship between FC of the NAc from “on” to “off” State and Pain Intensity

**Figure 2:**
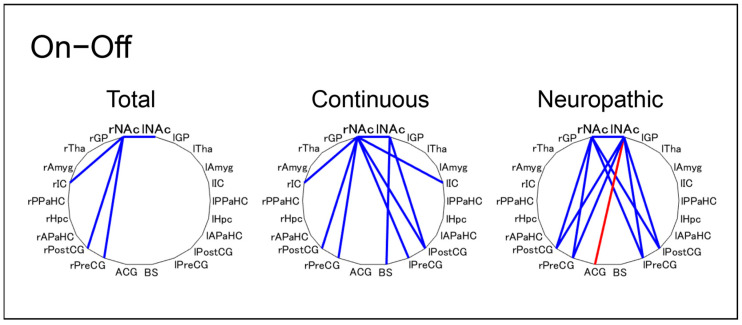
Characteristics of correlation coefficients between the SF-MPQ-2 in the “off” state and FC from “on” to “off” state. Blue lines indicate a significantly negative correlation (<-0.4) and red lines indicate a significantly positive correlation (>0.4). SF-MPQ-2: Japanese version of the revised short-form McGill Pain Questionnaire 2; FC: functional connectivity; r: right; l: left; NAc: Nucleus Accumbens; GP: Globus Pallidus; Tha: Thalamus; IC: Insular Cortex; Amyg: Amygdala; PPaHC: Posterior Parahippocampal Gyrus; Hpc: Hippocampus; APaHC: Anterior Parahippocampal Gyrus; PostCG: Postcentral Gyrus; PreCG: Precentral Gyrus; ACG: Anterior Cingulate Gyrus; BS: Brainstem.

We explored whether pain intensity in the “off” state is related to a change in FC from “on” to “off” state. Indeed, the SF-MPQ-2 total, continuous, and neuropathic subscale scores displayed a correlation with FC in the “off” state. Specifically, the SF-MPQ-2 total scores displayed a negative correlation with FC between the rNAc and rPreCG/rPostCG (*r* = −0.54 and −0.43, respectively) and between the rNAc and lNAc (*r* = −0.40). Regarding the subscales, continuous subscale scores displayed a correlation with FC of the rNAc with bilateral PreCG/PostCG and lNAc from “on” to “off” state, whereas neuropathic subscale scores displayed a correlation of FC between the bilateral NAc and bilateral PreCG/PostCG (Figure 2). Therefore, pain intensity in the “off” state was correlated with a change in FC between the NAc and primary motor/sensory cortices from “on” to “off” state.

### 3.4. Relationship between FC of the NAc from “on” to “off” State and Pain Developing from “on” to “off” State

**Figure 3:**
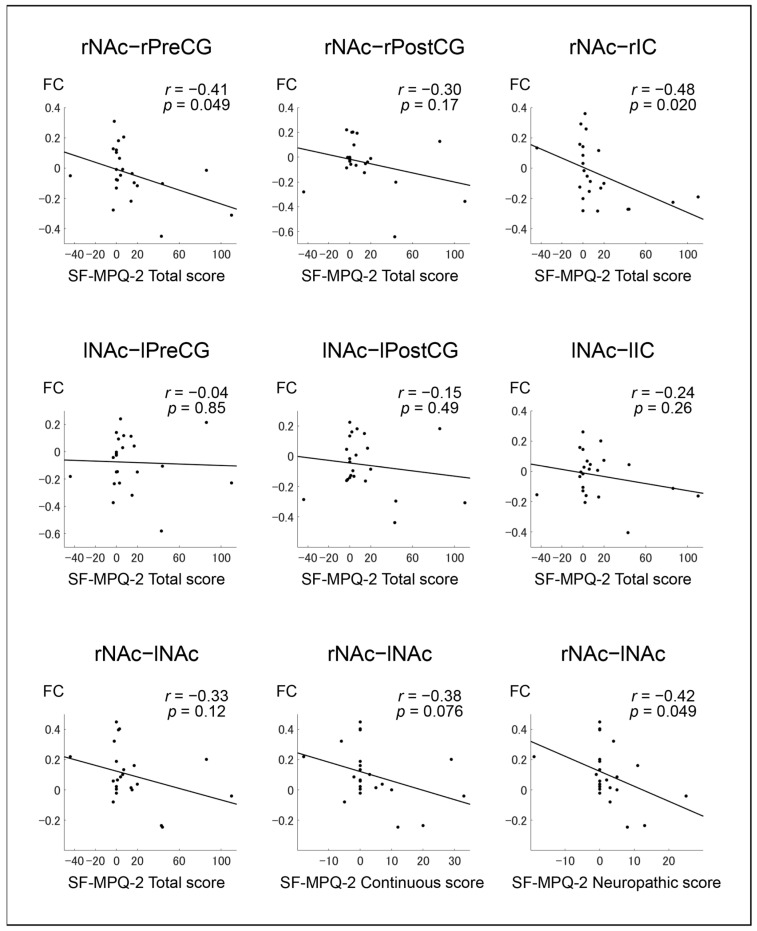
Pearson correlation coefficients and graphs for the correlation of SF-MPQ-2 from “on” to “off” state with FC from “on” to “off” state. The lines indicate the approximate line using the least squares method. SF-MPQ-2: Japanese version of the revised short-form McGill Pain Questionnaire 2; FC: functional connectivity; r: right; l: left; NAc: Nucleus Accumbens; PreCG: Precentral Gyrus; PostCG: Postcentral Gyrus; IC: Insular Cortex.

We conducted further analysis to explore whether the change in FC between the NAc and primary motor/sensory cortices in the ipsilateral hemisphere was related to pain intensity from “on” to “off” state. The changes in FC between the rNAc and rPreCG from “on” to “off” state displayed a negative correlation with the corresponding changes in the SF-MPQ-2 total (*r* = −0.41), continuous (*r* = −0.45), and neuropathic (*r* = −0.44) subscales (Figure 3 and Figure S1). The changes in FC between the rNAc and right insular cortex (rIC) from the “on” to “off” state showed a negative correlation with the changes in the scores of the SF-MPQ-2 total (*r* = −0.48) and neuropathic (*r* = −0.48) subscales (Figure 3 and Figure S1). The changes in FC of the right NAc and ipsilateral cerebral cortices, such as the PreCG and IC, were related to pain symptoms. Furthermore, the changes in FC from “on” to “off” state between the bilateral NAc displayed a negative correlation with the changes in the SF-MPQ-2 neuropathic subscale scores (*r* = −0.42; Figure 3). Taken together, aberrant changes in FC of the rNAc with the rPreCG/rIC and lNAc, from “on” to “off” state, were closely related to pain symptoms developing from “on” to “off” state.

## 4. Discussion

The results of current study revealed that aberrant FC associated with the NAc was related to pain symptoms, as both FC and pain symptoms were shown to change between the “on” and “off” states in PD.

As the disease progresses, patients with PD undergo treatment adjustments and show several motor complications, such as levodopa-induced dyskinesia, wearing-off phenomenon, and on-off fluctuations [23]. In PD, the on-off fluctuations not only influence the motor symptoms, but also the frequency and severity of non-motor symptoms, including pain [4,24,25]. The on-off fluctuations in non-motor symptoms were suggested to be related to dopaminergic stimulation and modulation of other neurotransmitter systems: glutamatergic, serotoninergic, and adrenergic [24]. The FC in brain networks is also altered between the “on” and “off” states, and this alteration in pain-related brain regions causes the fluctuation of pain observed in PD [7,8].

In previous studies, patients with PD showed more frequent and severe pain symptoms during the “off” state compared to the “on” state [4,24]. The loss of dopamine neurons in the substantia nigra pars compacta and the ventral tegmental area (VTA) leads to a reduced ability to regulate the release of dopamine, particularly during the “off” state [6,9]. In the mesolimbic dopaminergic pathway, the NAc receives dopamine from the VTA. The NAc, centered in the limbic circuit of the basal ganglia, plays an important role in pain processing, as well as in emotional learning, motivated and addictive behavior, and reward [5]. After receiving painful stimuli, the NAc releases opioids, which are regulated by dopamine from the VTA. Opioids decrease perception of painful stimuli by blocking pain signals from pain-related brain regions [6,9]. In the “off” state, PD-affected brains under central dopamine depletion show reduction of endogenous opioid release from the NAc, which results in patients reporting higher pain intensity compared with that experienced during the “on” state [4,26]. Indeed, our results were consistent with these findings, indicating that patients with PD have more severe pain intensity during the “off” state.

The mesolimbic pathway, which comprises dopaminergic neurons, plays an important role in pain processing. Dopaminergic neurotransmission in the mesolimbic pathway is impaired in patients with chronic pain [5,27], as well as depression and addiction, due to alteration in NAc plasticity [6]. The correlations between FC of the NAc with pain-related brain regions and pain intensity were different between the “on” and “off” states in this experiment. The changes in FC of the NAc in primary motor/sensory cortices from “on” to “off” state were correlated with pain intensity in the “off” state, and from “on” to “off” state. Previous studies have indicated that FC during the resting-state may be involved in PD-related pain [8,10]. Dopamine depletion is thought to be linked to pathological and compensatory changes in brain connectivity. During the dopaminergic “off” state, cortico-striatal hyperconnectivity is observed, and increased neuronal synchronization in the “off” state may reflect the compensatory response of non-dopaminergic systems and the network reorganization across the cortex and subcortex [7]. In an animal model of neuropathic pain, the FC of the NAc with the striatum and cortex were altered, and the disrupted NAc neuronal activity reduced the expression of neuropathic pain-related behavior [28]. Our results support previous findings showing that aberrant FC of the NAc with the cerebral cortex is correlated with the development of pain and its intensity from “on” to “off” state.

The current study provides novel insights into a potential mechanism underlying fluctuating pain in PD; however, several limitations should be considered. First, this clinical study was a pilot study with a limited sample size. In addition, since healthy subjects present less fluctuations in pain, we could not implement a proper control group of healthy subjects [29]. Therefore, a large clinical trial with an appropriate methodology and research design is needed to validate our findings. Second, we conducted a seed-based rsfMRI analysis by strictly using the bilateral NAc as seed regions because the present study focused on the NAc as a central component of pain processing. More detailed ROIs and whole-brain functional connectivity analyses are needed to elucidate the mechanisms of abnormal FC underpinning pain in PD. Additionally, we did not consider other clinical factors that might influence pain, such as mental orientation, depression, and sleep disorders. Indeed, the symptoms of depression have been suggested to be associated with aberrant connectivity in the default mode networks in patients with PD [30].

## 5. Conclusions

We conducted an rsfMRI analysis in patients with PD to explore the relationship between neuronal synchronization of the NAc and pain. The NAc in the mesolimbic pathway is suggested to be involved in the perception and modulation of pain symptoms [5,27]. Aberrant FC of the NAc with the cerebral cortex was related to the generation of pain from “on” to “off” state. These findings may facilitate our understanding of the mechanisms of abnormal connectivity underpinning pain development in patients with PD.

## Figures and Tables

**Table 1:** Demographic and clinical characteristics of the patients.

	PD (*n* = 23)		*p*-Value
Age	73.04 ± 7.96		
Sex	12 Male/11 Female		
Disease Duration, years	10.13 ± 6.68		
LEDD, mg	676.65 ± 272.55		
H-Y stages			
H-Y II	4 (17.4%)		
H-Y III	8 (34.8%)		
H-Y IV	9 (39.1%)		
H-Y V	2 (8.7%)		
MDS-UPDRS			
Total	On	Off	<0.05
	63.56 ± 31.55	77.48 ± 33.52	
Part I	13.91 ± 7.66		
Part II	16.61 ± 10.07		
Part III	On	Off	<0.05
	25.65 ± 15.38	40.35 ± 18.29	
Part IV	7.26 ± 3.92		
NRS	On	Off	0.068
	2.61 ± 1.83	2.87 ± 3.21	
SF-MPQ-2	On	Off	
Total	10.91 ± 23.14	24.83 ± 34.38	<0.05
Continuous	10.91 ± 23.14	24.83 ± 34.83	0.11
Intermittent	1.78 ± 4.24	5.22 ± 10.25	0.12
Neuropathic	2.70 ± 7.65	5.26 ± 8.62	0.12
Affective	2.26 ± 4.18	6.35 ± 9.85	<0.05
MMSE score	24.74 ± 3.83		
HDSR score	24.57 ± 4.33		
FAB score	12.61 ± 2.73		

Note: Data is represented by ±SD. Disease duration was calculated as the number of years since PD diagnosis. Hoehn and Yahr (H–Y) stages were used to assess the severity of motor symptoms. The MDS-UPDRS, NRS, and SF-MPQ-2 were administered during the “on” and “off” states at the time of the MRI examinations. PD: Parkinson’s disease; LEDD: levodopa equivalent daily dose; H-Y: Hoehn and Yahr; MDS-UPDRS: Movement Disorder Society Unified Parkinson’s Disease Rating Scale; NRS: Numerical Rating Scale; SF-MPQ-2: Japanese version of the revised short-form McGill Pain Questionnaire 2; MMSE: Mini-Mental State Examination; HDSR: Hasegawa Dementia Scale Revised; FAB: frontal assessment battery; SD: standard deviation.

## Data Availability

The data presented in this study are available on request from the corresponding author.

## Supplementary Materials

The following are available online at https://www.mdpi.com/article/10.3390/brainsci12010084/s1, Table S1: The 22 cortical and subcortical regions of interests (ROIs) used in this study; Figure S1: Pearson correlation coefficients and graphs indicating the correlation of SF-MPQ-2 from “on” to “off” state, with FC from the “on” to “off” state.
