# Supplementary material for: Pain-Related Abnormal Neuronal Synchronization of the Nucleus Accumbens in Parkinson’s Disease
Source: Brain Sci. 2022 Jan 7;12(1):84. doi: 10.3390/brainsci12010084 (PMC8773786; doi:10.3390/brainsci12010084)
Supplement: Supplementary file 1 [file brainsci-12-00084-s001.zip › brainsci-1497954-supplementary.pdf]

Table S1. The 22 cortical and subcortical regions of interests (ROIs) used in this study.

**Region**	**Abbreviation**
Right Nucleus Accumbens	rNAc
Left Nucleus Accumbens	lNAc
Right Globus Pallidus	rGP
Left Globus Pallidus	lGP
Right Thalamus	rTha
Left Thalamus	lTha
Right Insula Cortex	rIC
Left Insula Cortex	lIC
Right Amygdala	rAmyg
Left Amygdala	lAmyg
Right Posterior Parahippocampal Gyrus	rPPaHC
Left Posterior Parahippocampal Gyrus	lPPaHC
Right Hippocampus	rHpc
Left Hippocampus	lHpc
Right Anterior Parahippocampal Gyrus	rAPaHC
Left Anterior Parahippocampal Gyrus	lAPaHC
Right Postcentral Gyrus	rPostCG
Left Postcentral Gyrus	lPostCG
Right Precentral Gyrus	rPreCG
Left Precentral Gyrus	lPreCG
Anterior Cingulate Gyrus	ACG
Brainstem	BS

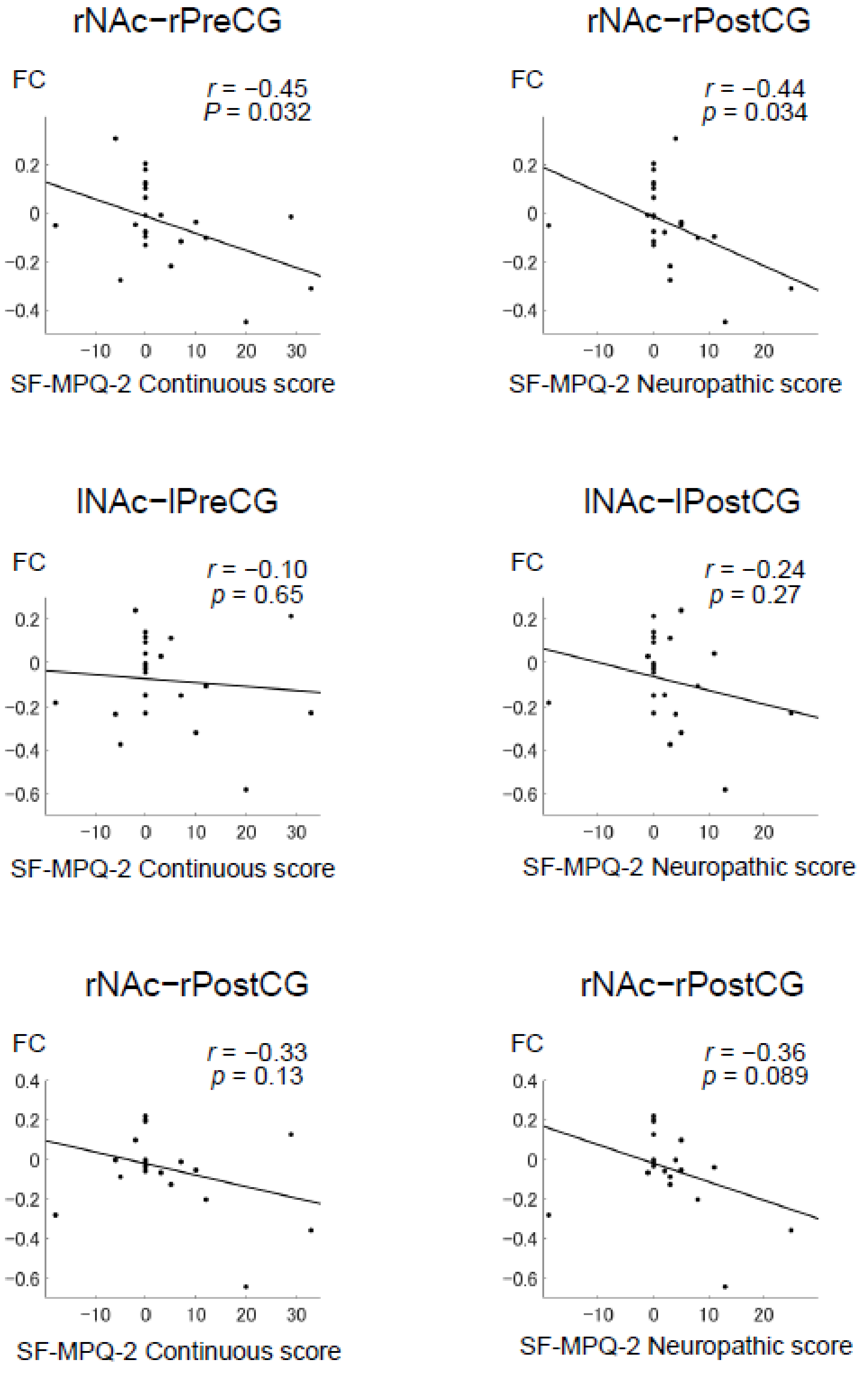

rNAc−rPreCG
FC
r = −0.45
P = 0.032
SF-MPQ-2 Continuous score
rNAc−rPostCG
FC
r = −0.44
p = 0.034
SF-MPQ-2 Neuropathic score
lNAc−lPreCG
FC
r = −0.10
p = 0.65
SF-MPQ-2 Continuous score
lNAc−lPostCG
FC
r = −0.24
p = 0.27
SF-MPQ-2 Neuropathic score
rNAc−rPostCG
FC
r = −0.33
p = 0.13
SF-MPQ-2 Continuous score
rNAc−rPostCG
FC
r = −0.36
p = 0.089
SF-MPQ-2 Neuropathic score

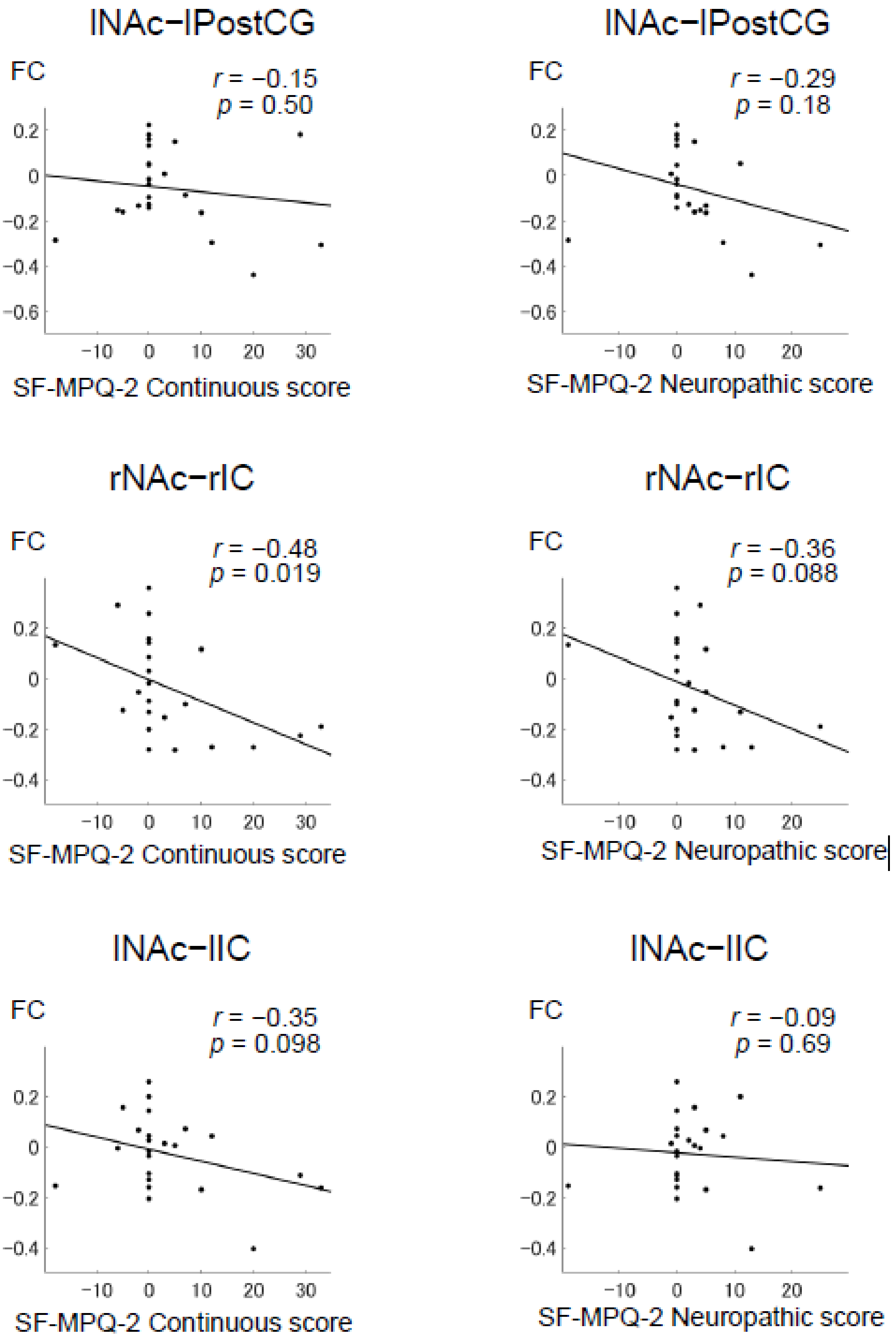

Figure S1: Pearson correlation coefficients and graphs indicating the correlation of SF-MPQ-2 from "on" to "off" state, with FC from the "on" to "off" state. The lines indicate the approximate line using the least squares method. SF-MPQ-2: Japanese version of the revised short-form McGill Pain Questionnaire 2; FC: functional connectivity; r: right; l: left; NAc: Nucleus Accumbens; PreCG: Precentral Gyrus; PostCG: Postcentral Gyrus; IC: Insula Cortex.
